# Dementia spectrum disorders: lessons learnt from decades with PET research

**DOI:** 10.1007/s00702-019-01975-4

**Published:** 2019-02-14

**Authors:** Heather Wilson, Gennaro Pagano, Marios Politis

**Affiliations:** Neurodegeneration Imaging Group, Maurice Wohl Clinical Neuroscience Institute, 125 Coldharbour Lane, Camberwell, London, SE5 9NU UK

**Keywords:** Dementia, Alzheimer’s disease, Parkinsonism dementias, Molecular imaging, Positron emission tomography

## Abstract

The dementia spectrum encompasses a range of disorders with complex diagnosis, pathophysiology and limited treatment options. Positron emission tomography (PET) imaging provides insights into specific neurodegenerative processes underlying dementia disorders in vivo. Here we focus on some of the most common dementias: Alzheimer’s disease, Parkinsonism dementias including Parkinson’s disease with dementia, dementia with Lewy bodies, progressive supranuclear palsy and corticobasal syndrome, and frontotemporal lobe degeneration. PET tracers have been developed to target specific proteinopathies (amyloid, tau and α-synuclein), glucose metabolism, cholinergic system and neuroinflammation. Studies have shown distinct imaging abnormalities can be detected early, in some cases prior to symptom onset, allowing disease progression to be monitored and providing the potential to predict symptom onset. Furthermore, advances in PET imaging have identified potential therapeutic targets and novel methods to accurately discriminate between different types of dementias in vivo. There are promising imaging markers with a clinical application on the horizon, however, further studies are required before they can be implantation into clinical practice.

## Introduction

Neurodegenerative dementia spectrum disorders are associated with progressive and irreversible neuronal loss. Dementia encompasses a wide range of disorders which are often difficult to accurately diagnose and there are limited treatments options. Positron emission tomography (PET) is a molecular imaging technique which has the potential to aid understanding of disease pathophysiology, differential diagnoses between dementia disorders, monitor disease progression and response to treatments (Politis et al. 2017; Rocchi et al. 2015; Politis and Piccini 2012; Politis 2014). The development of new PET tracers provides increasing ability to visualise distinct histopathologic hallmarks underlying different dementias (Table 1). Other neuroimaging techniques such as magnetic resonance imaging (MRI) have also been used to investigate dementia pathophysiology including regional brain atrophy, changes in white matter integrity and functional connectivity. This review provides an overview of advances in PET imaging across common dementias.

**Table 1:** PET tracers employed to assess pathophysiology in dementia spectrum disorders

Target system	PET radioligand	Disease cohort	PET molecular changes	Applications
Brain glucose metabolism	[^18^F]FDG	AD	Hypometabolism in the parietotemporal, posterior cingulate, medial temporal, with additional hypometabolism in the frontal cortex in advanced AD Hypometabolism is associated with cognitive decline	Differential diagnosis between Parkinsonian dementia (PDD or DLB) and dementia due to AD
		DLB	Widespread hypometabolism with most prominent metabolic reductions in the occipital cortex	
		PDD	Greater hypometabolism in the visual cortex and persevered metabolism in medial temporal cortex compared to AD	
		PSP	Hypometabolism in the medial frontal cortex, premotor areas prefrontal areas, striatum (predominantly in the caudate), thalamus, and brainstem	Differential diagnosis between PD and atypical parkinsonism (MSA, PSP or CBS)
		CBS	Asymmetric hypometabolism contralateral to the clinically most affected side involving parietal cortex, primary sensorimotor cortex, the medial and lateral premotor areas, striatum, and thalamus	
		MSA	Hypometabolism in the putamen, cerebellum, and brainstem Specific patterns depending of the subtype; MSA-P have pronounced hypometabolism in the putamen, while MSA-C has more pronounced hypometabolism in cerebellar hemispheres and middle cerebellar peduncle	
		FTD	bv FTD: frontal lobe hypometabolism accompanied by temporal and subcortical hypometabolism in advanced stages	Differential diagnosis between FTD and AD
Amyloid-β pathology	[^11^C]PIB [^18^F]AV45 (also known as [^18^F]Florbetapir) [^18^F]AZD4694 (also known as [^18^F]NAV4694) [^18^F]Florbetaben [^18^F]Flutemetamol	AD	High cortical uptake, mostly in frontal parietal, and temporal association cortices Predictive of cognitive deterioration in patients with MCI and probably AD	Clinical diagnosis of AD, based on positive amyloid load; and differential diagnosis between FTD and AD
		DLB	High cortical [^11^C]PiB retention associated with cognitive impairment	Differentiate DLB from PDD
		PDD	Lower cortical [^11^C]PiB retention compared to DLB. Amyloid-β positive PDD is associated with increased amyloid-β in cortical and striatal regions and could be predictive of cognitive decline	
		PSP	Normal	
		CBS	Normal	
		MSA	Normal	
		FTD	Low cortical [^11^C]PIB retention in line with controls, supporting the absence of amyloid-β pathology in FTD	Differential diagnosis between FTD and AD
Tau pathology	[^18^F]AV1451 (also known as [^18^F]T807) [^18^F]THK5105 [^18^F]THK5117 [^18^F]THK5351 [^18^F]THK523 [^11^C]PBB3 [^18^F]FDDNP [^18^F]PI-2620 [^18^F]MK-6240	AD	[^18^F]AV1451 uptake is consistent with the known distribution of tau pathology, and compatible with Braak staging Increased [^18^F]AV1451 uptake is associated with dementia severity and cognitive impairment	To monitor disease progression and conversion from MCI to AD
		DLB	Increased [^18^F]AV1451 uptake has been reported in the posterior temporoparietal, occipital cortex and precuneus in DLB	Increased [^18^F]AV1451 uptake in the medial temporal cortex could distinguish AD dementia from probable DLB
		PDD	Increased [^18^F]AV1451 in inferior temporal cortex compared to PD without cognitive impairment. Lower tau load in the cortex and striatum compared to DLB	Preliminary results from second-generation tau tracers, such as [^18^F]PI-2620, suggest different and specific binding patterns, with no off-target binding, indicating the potential of tau PET imaging as a tool to aid differential diagnosis
		PSP	Increased [^18^F]FDDNP binding in subthalamic area, midbrain region, and cerebellar white matter. Increased [^18^F]FDDNP binding in neocortical regions (frontal lobe, temporal lobe an posterior cingulate gyrus) only in PSP subjects with more severe disease	
		CBS	Increased [^18^F]AV1451 uptake, in the absence of amyloid, in frontal and parietal cortical regions in CBS Higher [^18^F]THK5351 uptake in frontal, parietal and the globus pallidus in CBS patients Both [^18^F]AV1451 and [^18^F]THK5351 showed higher binding contralateral to the clinically most affected side	
		MSA	Tau pathology should be absent, however, MSA patients with significant glial cytoplasmic inclusion burden can result in a false positive on tau PET imaging. Increased uptake of [^18^F]AV1451 in posterior putamen and increased uptake of [^11^C]PBB3 in cortical and subcortical regions has been described	
		FTD	Increased [^18^F]AV1451 binding in frontal, insular, anterior temporal and cingulate cortices in FTD Increased [^18^F]FDDNP uptake has also been reported in frontal and lateral temporal regions in FTLD bv FTD showed increased [^18^F]AV1451 in the anterior temporal lobes and anterior cingulate cortex	FTLD uptake in the parietal cortex is lower than in AD and could aid differential diagnosis of FTLD from AD
Neuroinflammation (TSPO)	[^11^C]PK11195 [^11^C]PBR28 [^18^F]PBR06 [^18^F]PBR111 [^18^F]FEPPA [^11^C]DAA1106 [^18^F]FEDAA1106	AD	Increased [^11^C]PK11195 binding in frontal, temporal, parietal, occipital and cingulate cortices in AD, with similar distribution pattern to amyloid-β plaques Association between microglial activation and cognitive impairment	Potential to monitor disease severity and cognitive decline
		DLB	Increased [^11^C]PK11195 binding in cortex, basal ganglia and substantia nigra Higher cortical [^11^C]PK11195 binding correlated with cognitive impairment	Potential to monitor cognitive decline
		PDD	Increased [^11^C]PK11195 binding in the association cortex in PDD compared to non-demented PD Increased microglia activation in the cingulate, frontal, temporal and occipital cortical regions, as well as the striatum Association between microglial activation and cognitive impairment	
		PSP	Increased [^11^C]PK11195 binding in caudate, putamen, pallidum, substantia nigra, midbrain, thalamus, cerebellum, and frontal lobe	Potential to monitor anti-inflammatory or immunomodulatory therapies *in vivo*
		CBS	Increased [^11^C]PK11195 binding in caudate, putamen, substantia nigra, and frontoparietal cortex	
		MSA	Increased [^11^C]PK11195 binding was also found in the dorsolateral prefrontal cortex, caudate, putamen, pallidum, thalamus, substantia nigra, and pons	
		FTD	Increased [^11^C]PK11195 binding in FTD in the frontotemporal regions; with greater uptake in frontal subcortical white matter in FTD compared to AD	
Cholinergic system	[^11^C]PMP (Presynaptic AChE) [^11^C]MP4A (Presynaptic AChE) [^18^F]FEOBV (Presynaptic VAChT) [^11^C]nicotine (Postsynaptic nAChR) [^18^F]2FA (Postsynaptic α4β2 nAChR) [^18^F]AZAN (Postsynaptic α4β2 nAChR) [^11^C]NMPB (mAChR)	AD	Reduced AChE activity in the cortex, hippocampus and amygdala consistent with widespread ChAT and AChE loss observed in AD post-mortem studies Cortical cholinergic deficits are associated with increased severity of cognitive impairment	To monitor cognitive decline and assess the effectiveness of new therapeutic treatments
		DLB	Decreased cortical AChE activity in DLB compared to AD No differences in AChE density between DLB and PDD	
		PDD	Loss of cortical AChE activity is more apparent in PDD than in non-demented PD. Lower cortical AChE activity was associated with cognitive deficits but not with severity of motor symptoms	
		PSP	No significant changes in cortical mAChR levels in PSP patients with cognitive impairment Decreased subcortical AChE activity with greater involvement of the pontine cholinergic group	Differing cortical AChE activity in PSP and PD indicates potential use of to aid differential diagnosis
		CBS	Decreased levels of AChE in cortical regions including the paracentral, frontal, parietal and occipital cortex	To assess the effectiveness of new therapeutic treatments
		MSA	Decreased cortical and subcortical AChE activity in MSA-P	
		FTD	No differences in AChE activity in FTD compared to healthy controls	Potential use of to aid differential diagnosis

*TSPO* translocator protein, *ChAT* choline acetyltransferase, *AChE* acetylcholinesterase, *VAChT* vesicular acetylcholine transporter, *mAChR* muscarinic acetylcholine receptors, *nAChRs* nicotinic acetylcholine receptors, *AD* Alzheimer’s disease, *MSA* Multiple system atrophy, *MSA-P* Parkinsonian type multiple system atrophy, *MSA-C* Cerebellar ataxia type multiple system atrophy, *PSP* Progressive supranuclear palsy, *CBS* Corticobasal syndrome, *DLB* dementia with Lewy bodies, *PDD* Parkinson’s disease dementia, *bvFTD* behavioural variant of Frontotemporal dementia

Dementia disorders can be classified according to their pathology of misfolded proteins including amyloid-β, tau and alpha-synuclein. Alzheimer’s disease (AD), the most common neurodegenerative dementia disorder, is pathologically characterised by extracellular amyloid plaques composed of amyloid-β, and intracellular neurofibrillary tangles (NFTs) composed of hyperphosphorylated tau protein (Hardy 2006). AD usually presents with difficulties remembering autobiographic events and to a lesser extent by problems with language, executive and visuospatial functions (McKhann et al. 2011). Mild cognitive impairment (MCI) can represent a transitional stage between healthy and AD with 12–15% progressing from MCI to AD each year (Petersen et al. 2009). MCI patients present with mild memory impairments on cognitive testing but are often not affecting their daily function.

Frontotemporal lobe degeneration (FTLD) is associated with aggregated tau, as Pick bodies, and encompasses several distinct pathologies involving frontal and/or anterior temporal lobe degeneration alongside associated dementia (Rabinovici and Miller 2010). Frontotemporal lobar degenerative disorders include behavioural frontotemporal dementia (FTD), semantic dementia and progressive non-fluent aphasia (Olney et al. 2017).

Degenerative parkinsonian disorders can be broadly divided into two groups based on neuropathological characteristics: (1) synucleinopathies, which includes PD with dementia (PDD), dementia with Lewy bodies (DLB) and multiple system atrophies (MSAs); and (2) tauopathies, including progressive supranuclear palsy (PSP) and corticobasal syndrome (CBS), associated with intra-neuronal and astrocytic aggregates of tau. The neuropathology of synucleinopathies is characterised by the presence of alpha-synuclein, which form the main component of Lewy bodies and neurites (Spillantini et al. 1997). Histopathological evidence suggests that alpha-synuclein often coexists with amyloid-β plaques and tau neurofibrillary tangles (Compta et al. 2014; Horvath et al. 2013). Such neuropathological changes could result in disruption of the dopaminergic, cholinergic and noradrenergic neuromodulating projecting networks as well as local or regional neuronal and synaptic dysfunction. In vivo molecular imaging tools could help to elucidate the complex interaction between PD-like (alpha-synuclein) and AD-like (amyloid-β plaques and tau) pathologies and their role in the development of cognitive impairment.

Generally cognitive deficits in parkinsonian dementia syndromes relate aberrant information processing compared to AD which typically involves more content-specific deficits in language and perceptual processing. Dementia is common in PD patients with over 80% of patients developing dementia after 20 years of the disease (Aarsland et al. 2008, 2017). PDD and DLB are clinically distinguished based only on whether Parkinsonism or dementia developed first (McKeith et al. 2005; Niccolini and Politis 2016). Patients with levodopa-responsive parkinsonism who develop dementia more than 1 year after the onset of cardinal parkinsonism motor symptoms are classified as PDD; while patients who develop dementia and parkinsonism within 1 year or when the onset of dementia precedes parkinsonism are classified as DLB.

## Glucose metabolism: FDG PET imaging

[^18^F]fluorodeoxyglucose (FDG) PET studies are used to estimate the local cerebral metabolic rate of glucose consumption; providing information on the distribution of neuronal death and synaptic dysfunction with relatively disease-specific reduction patterns (Magistretti 2000).

### FDG-PET in Alzheimer’s disease

FDG–PET plays a key role in early and differential diagnosis of AD due to unique patterns of hypometabolism (reduced FDG uptake). Retrospective studies have illustrated FDG–PET has 94% sensitivity and 73% specificity to predict AD pathology (Silverman et al. 2001). In early stages of AD, FDG–PET has revealed hypometabolism in the parietotemporal association cortices, posterior cingulate and precuneus regions (Minoshima et al. 1995). Hypometabolic regions spread to the frontal association cortices in moderate-to-severe AD, while metabolism in the striatum, thalamus, primary sensorimotor cortices, visual cortices and cerebellum are relatively persevered through disease progression. Compared to late-onset AD, patients with early-onset experience greater hypometabolism in parietal and posterior cingulate cortices and precuneus regions to reach the same severity of dementia (Kim et al. 2005). Lower FDG uptake is associated with lower cognitive function measures such as the Mini-Mental State Examination (MMSE) and specific word recognition tests (Landau et al. 2011). Moreover, glucose metabolism has been suggested as a sensitive marker for predicting future cognitive decline (Landau et al. 2011; Zhang et al. 2012; Arbizu et al. 2013). Recently, it has been reported that hypertension in AD patients is associated with worse cognitive function and reduced glucose hypometabolism in the hippocampus (Moonga et al. 2017).

There is evidence to suggest changes in glucose metabolism occur early in preclinical AD stages. In autosomal dominant AD, patterns of hypometabolism presenting up to 10 years before symptom onset are consistent with patterns observed in established AD (Kennedy et al. 1995; Bateman et al. 2012). Similar patterns were also observed in cognitively normal apolipoprotein (APOE) e4 carriers who have an increased risk of developing AD (Langbaum et al. 2010; Knopman et al. 2014). FDG-PET studies in MCI have demonstrated similar patterns of glucose reduction as in early AD, with very early metabolic deficits in the medial portion of the parietal cortex, posterior cingulate and spreading to temporal and prefrontal cortices (Small et al. 2008). FDG–PET studies have shown patterns of hypometabolism in the inferior parietal lobe, precuneus and posterior cingulate can predict the conversion from MCI to AD (Yuan et al. 2009). Moreover, FDG–PET was found to have higher accuracy for predicting conversion of MCI to AD than structural MR imaging (Yuan et al. 2009).

Multimodal imaging studies, using FDG PET and amyloid PET have investigated the relationship between amyloid plaque deposition and glucose metabolism. These studies have produced conflicting results with some showing an association between local amyloid plaque load and hypometabolism (Lowe et al. 2014; Engler et al. 2006; Cohen et al. 2009; Edison et al. 2007) and others showing no correlation (Li et al. 2008; Furst et al. 2012; Rabinovici et al. 2010). Discrepancies in findings could be due to changing relationship between amyloid plaque load and glucose metabolism depending on disease stage or brain region. A recent study suggests cortical hypometabolism is mainly linked to increased global, rather than regional, amyloid burden (Altmann et al. 2015). Further longitudinal studies are required to clarify mechanisms underlying the relationship between glucose metabolism and amyloid plaque load.

### FDG-PET in parkinsonian dementias

Patterns of hypometabolism in DLB are similar to those observed in AD, with early involvement of temporal lobes extending to parietal and frontal areas. However, in DLB reduced metabolism also occurs in the occipital primary and association areas (Minoshima et al. 2001; Ishii et al. 1998). Patients with PDD showed greater metabolic reduction in the visual cortex and persevered metabolism in medial temporal cortex compared to AD patients (Vander Borght et al. 1997). Therefore, occipital hypometabolism is a marker to distinguish between patients with PDD or DLB and AD with 90% sensitivity and 80% specificity (Minoshima et al. 2001). Decreased metabolic activity in the anterior cingulate is potentially greater in DLB than in PDD (Yong et al. 2007); however, clear differentiation between PDD and DLB has proven difficult due to mostly similar metabolic patterns. Therefore, while FDG–PET can aid differential diagnosis between parkinsonian dementia (PDD or DLB) and dementia due to AD, is not able to clearly differentiate between PDD and DLB.

Combined PET imaging of amyloid-β plaques and FDG showed parietotemporal and occipital metabolism was significantly lower in patients with DLB compared to AD (Ishii et al. 2015; Kantarci et al. 2012). Furthermore, there was no difference in regional glucose metabolism between amyloid-β positive and amyloid-β negative DLB patients. This suggests that regional hypometabolism in DLB is independent of AD amyloid-β pathology, with hypometabolism is much higher in DLB than AD (Ishii et al. 2015; Kantarci et al. 2012; Fujishiro et al. 2010). Therefore, the presence of hallmark alpha-synuclein pathology could contribute to the majority of cognitive decline and regional hypometabolism in DLB.

In early stages of PSP, decreased glucose metabolism in the midbrain, lateral and medial frontal lobes and caudate nucleus has been reported (Juh et al. 2005, 2004) in FDG-PET studies which can distinguish PSP from other parkinsonism diseases (Hosaka et al. 2002; Klein et al. 2005). CBS patients with symptoms of dementia show an AD-like pattern of hypometabolism but with asymmetric glucose metabolic reduction in the lateral frontal, lateral temporal and medial and lateral parietal cortices, the pre- and post-central gyri and thalamus (Hirono et al. 2000). A comparison between CBS and PSP revealed relative hypometabolism in the thalamus and mid-brain in PSP patients and the parietal lobe in CBS patients (Juh et al. 2005).

### FDG-PET in frontotemporal lobar degenerative disorders

In FTD, frontal and temporal regions, as well as the striatum and thalamus show decreased glucose metabolism (Ishii et al. 1998a, b). Metabolic and morphological changes occur in bilateral frontal and temporal lobes, whereas regions of metabolism are more severely affects than regions of atrophy in the frontal lobe (Engler et al. 2008). As the disease progresses to advanced stages, hypometabolism spreads from localised frontal lobe areas to the parietal and temporal cortices (Diehl-Schmid et al. 2007). FDG-PET can be used to aid clinical diagnosis of FTLD, using a visual rating scale of cerebral metabolism, with a sensitivity of 89% (Poljansky et al. 2011). FTD and semantic dementia present with distinct patterns of cerebral metabolism. FTD is associated with frontal hypometabolism, whereas patients with semantic dementia have severe hypometabolism in the temporal lobe (Diehl et al. 2004). Furthermore, patients with FTLD have significantly lower metabolism in the frontal lobe and basal ganglia compared to AD and MCI (Poljansky et al. 2011). Therefore, FDG-PET could be used to differentiate between diagnosis of FTLD, AD and MCI.

## Amyloid PET imaging

[^11^C]Pittsburgh compound-B (PIB) has been the most widely used PET tracer for amyloid imaging over the past decade. However, the short half-life of [^11^C]PIB, requiring an onsite cyclotron and synthesis system, limits its clinical use. Therefore, second generation ^18^F-labelled amyloid tracers, with longer half-life, such as ^18^F-AV-45 (florbetapir) (Okamura and Yanai 2010) and [^18^F]AZD4694 (Cselenyi et al. 2012), [^18^F]florbetaben (Fodero-Tavoletti et al. 2012) and [^18^F]flutemetamol (Vandenberghe et al. 2010), have greater potential to aid accurate diagnosis and evaluation of disease-modifying therapies in dementia disorders. PIB and ^18^F-labelled PET tracers bind with high affinity to the β sheet structure of fibrillary amyloid; thus, binding specifically to amyloid-β peptide aggregates and not other misfolded proteins, such as tau or α-synuclein (Fodero-Tavoletti et al. 2012).

### Amyloid imaging in Alzheimer’s disease

PIB–PET studies have demonstrated PIB retention consistent with post-mortem distribution of amyloid-β plaques (Ikonomovic et al. 2008). In clinical practice, visual analysis of amyloid PET provides binary information on the presence or absence of amyloid load in individuals with high sensitivity and specificity (Teipel et al. 2015). Longitudinal studies have shown that PIB–PET is able to detect the presence of amyloid-β plaques many years before the clinical disease onset in autosomal dominant AD (Benzinger et al. 2013). PIB has also been shown to predict cognitive decline in healthy individuals and patients with amnestic mild cognitive impairment (aMCI) (Okello et al. 2009; Lim et al. 2014). Furthermore, [^18^F]florbetapir has been shown to be predictive of cognitive deterioration over a 3 year follow up in patients with MCI and probably AD (Doraiswamy et al. 2014). Disease-specific PET tracers currently available for clinical use to distinguish AD and MCI include tracers for amyloid-β plaques to show or exclude brain amyloid load; [^18^F]florbetapir, [^18^F]florbetaben and [^18^F]flutemetamol.

According to the amyloid cascade hypothesis, the accumulation of amyloid-β is an early preclinical pathological process preceding cognitive decline (Jack et al. 2010). While amyloid imaging may play a role in diagnosis of AD, amyloid PET imaging does not correlate with cognitive decline and is weak in monitoring progression once early AD is established (Engler et al. 2006). Moreover, amyloid-β immunotherapies do not suppress dementia progression unless the drug also diminishes tau pathology (Karran and Hardy 2014). Thus, amyloid deposition may have already peaked in a patient with MCI and reached a plateau by early AD. Amyloid deposition is also not unique to AD and can be found in healthy elderly people. [^11^C]PIB PET has shown approximately one-third of healthy elderly individuals show increased amyloid deposition (Pike et al. 2007; Aizenstein et al. 2008). Therefore, clinical impairment may be due to, in part, the ability of individuals to tolerate aggregated amyloid. Genetic risk factors, lifestyle choices, environmental factors and neuropathological comorbidities could alter the threshold for the onset of cognitive impairment associated with amyloid-β aggregation (Bennett et al. 2006; Schneider et al. 2009). Studies have shown that APOE genotype differentially effects the distribution of amyloid plaques in AD (Ossenkoppele et al. 2013). The prevalence of amyloid pathology in people without dementia is associated with age, APOE genotype and presence of cognitive impairment. Moreover, it is suggested that there is a 20- to 30-year interval between first development of amyloid positivity and onset of dementia (Ossenkoppele et al. 2015; Jansen et al. 2015).

Going forward, the role of amyloid PET in clinical practice is in early stages of dementia to predict, on an individual basis, the risk of disease conversion from MCI to AD. However, amyloid PET alone is unlikely sufficient to accurately represent timings MCI to AD conversion.

### Amyloid imaging in parkinsonian dementia

Amyloid deposits, assessed with PIB-PET, have been associated with AD-like atrophy in patients with DLB and PDD (Shimada et al. 2013). Amyloid deposition occurs early in DLB and may account in for the early presentation of dementia. Amyloid burden is higher in patients with DLB compared to PDD, but not as high as PIB levels observed in AD (Fujishiro et al. 2010; Gomperts et al. 2008). PDD may be differentiated from DLB by its relatively lower amyloid plaque load (Brooks 2009). PIB binding has been linked to cognitive impairment in DLB (Gomperts et al. 2012). In PDD typically PIB binding does not differ from PD patients or healthy individuals (Gomperts et al. 2008). However, cortical PIB retention has been shown to predict cognitive decline in PD (Gomperts et al. 2016). Therefore, amyloid deposition may contribute to the timing of dementia onset in Lewy body disorders.

### Amyloid imaging in frontotemporal lobar degenerative disorders

PIB PET studies have revealed FTD patient’s exhibit no PIB retention; therefore, supporting PIB PET as a tool to differentiate between FTD and AD (Engler et al. 2008; Rabinovici et al. 2007). The identification of PIB-positive patients with frontotemporal lobe degeneration could suggest a mimicking of AD pathology. Furthermore, amyloid PET imaging can also be used to differentiate the diagnosis of semantic dementia from AD (Drzezga et al. 2008).

## Tau imaging

Advances in the development of PET tracers selective for tau have, for the first time, enabled in vivo evaluation of tau pathology in dementias. First generation tau selective PET tracers include [^18^F]AV1451 (also known as [^18^F]T807) (Xia et al. 2013; Marquie et al. 2015) and THK series: [^18^F]THK523 (Fodero-Tavoletti et al. 2011), [^18^F]THK5105 (Okamura et al. 2014), [^18^F]THK5117 (Harada et al. 2015; Jonasson et al. 2016), [^18^F]THK5351 (Harada et al. 2016). These tau tracers bind to NFTs with higher affinity than amyloid-β fibrils (Fodero-Tavoletti et al. 2011). However, first generation tau tracers have limitations due to off-target binding to monoamine oxidase (MAO)-A/B. Furthermore, [^18^F]AV1451, [^18^F]THK5117 and [^18^F]THK5105 have substantial retention in striatal regions even in cognitively normal subjects which is inconsistent with neuropathological reports and could lead to overestimation of tau burden in adjacent cortical areas (Serrano-Pozo et al. 2011). First generation tau PET tracers predominantly detect AD like tau pathology, paired helical filaments with a mixture of 3-repeat (3R) and 4-repeat (4R) tau isoforms. The ability of [^18^F]AV1451 to detect 4R-tau isoforms, predominant in CBS and PSP, has been debated with autoradiography studies showing lower binding of [^18^F]AV1451 in non-AD brains compared to AD (Marquie et al. 2015; Sander et al. 2016; Lowe et al. 2016).

Advances in novel second-generation tau radioligands, [^18^F]PI-2620 and [^18^F]MK-6240, with no off-target binding have greater potential to aid accurate diagnosis and evaluation of disease-modifying therapies. [^18^F]MK-6240 has higher affinity for neurofibrillary tangles in AD brains than [^18^F]AV1451, greater uptake contrast between the hippocampus and cortical regions and no off-target binding to MAO-A/B (Hostetler et al. 2016; Walji et al. 2016; Collier et al. 2017; Lohith et al. 2016). Preliminary results from [^18^F]PI-2620 are promising with low affinity for MAO-A/B and strong binding to both tau isoforms, 3R-tau in Pick’s disease and 4R-tau in PSP (Mueller et al. 2017; Barret et al. 2017). Thus, with specificity for 3R and 4R tau, [^18^F]PI-2620 has the potential to quantify tau pathology in vivo in AD patients as well as other dementias with prominent 4R-tau pathology such as PSP and CBS. Advances in new tau PET tracers provide opportunities to investigate regional distribution of tau pathology, clinical relevance to dementia and parkinsonian disorders, support early differential diagnosis and monitoring disease progression.

### Tau imaging in Alzheimer’s disease

Unlike amyloid-β deposition which accumulate early in AD and plateaus by symptom onset, intracellular neurofibrillary tangles (NFT) formation starts later and spreads throughout the course of the disease (Serrano-Pozo et al. 2011). Moreover, neuropathological studies show accumulation of NFT correlates with neuronal dysfunction and symptom progression (Serrano-Pozo et al. 2011; Arriagada et al. 1992; Bierer et al. 1995a, b). Distinct timing and distribution of amyloid-β plaques and tau suggests they aggregate independent, however, their pathological relationship is complex (Ittner and Gotz 2011). It is hypothesised that tau pathology is more closely linked to symptomatology and patterns of glucose hypometabolism in AD.

**Fig. 1:**
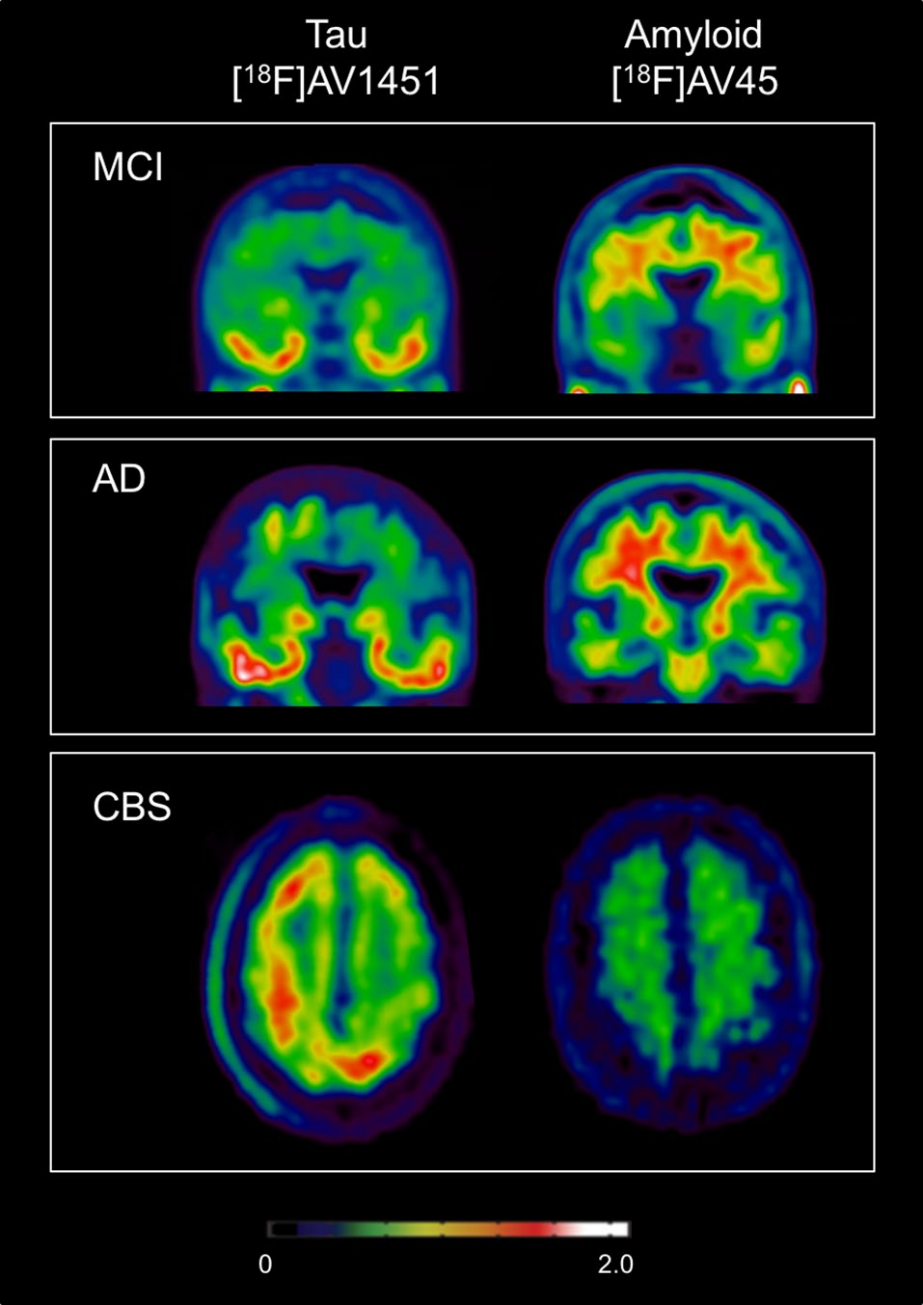
PET with [^18^F]AV1451 and [^18^F]AV45 showing differentiate uptake patterns of tau and amyloid pathology respectively in Mild Cognitive Impairment (MCI), Alzheimer’s Disease (AD) and Corticobasal Syndrome (CBS). MCI and AD subjects show increased [^18^F]AV1451 in temporal cortices, while CBS subject shows asymmetrical uptake in frontal and parietal regions. MCI and AD show positive [^18^F]AV45 scans with widespread amyloid binding while CBS subject has no amyloid pathology. MCI patient 77 years of age, MMSE = 28, MoCA = 15; AD patient 75 years of age, MMSE = 25, MoCA = 15; and CBS patients 75 years of age, MMSE = 17, MoCA = 15. *AD* Alzheimer’s Disease, *CBS* Corticobasal Syndrome, *MCI* Mild Cognitive Impairment, *MMSE* Mini-Mental State Examination, *MoCA* Montreal Cognitive Assessment

Early PET studies with [^18^F]THK523 showed higher retention in temporal, parietal and orbitofrontal cortices and in the hippocampus in AD patients compared to healthy controls (Villemagne et al. 2014). However, high white matter retention of [^18^F]THK523 hinders accurate visual interpretation of signals, thus preventing use in research or clinical settings (Villemagne et al. 2014). [^18^F]THK5105 and [^18^F]THK5117 showed higher binding affinity than [^18^F]THK523 (Okamura et al. 2005). PET studies with improved derivatives, [^18^F]THK5105 and [^18^F]THK5117, revealed binding in the temporal lobe clearly differentiating AD patients from healthy elderly subjects, and distinct from amyloid-β PET imaging with PIB (Okamura et al. 2014; Harada et al. 2015). Furthermore, [^18^F]AV1451 PET demonstrated cortical retention consistent with the known distribution of tau (Xia et al. 2013; Arnold et al. 1991; Schwarz et al. 2016) and a strong association with dementia severity and cognitive impairment (Ossenkoppele et al. 2016; Cho et al. 2016) (Fig. 1). In vivo studies show [^18^F]AV1451 uptake in AD patients is compatible with Braak staging (Schwarz et al. 2016). Therefore, supporting evidence from post-mortem and animal studies suggesting that aggregation of tau is closely linked to patterns of neurodegeneration and clinical manifestations of AD.

The use of PET as a tool to quantify tau pathology has been investigated to monitor conversion from MCI to AD. [^18^F]THK5117, [^18^F]THK5351 and [^18^F]AV1451 have shown highest uptake in AD followed by MCI, compared with low signal in normal controls (Harada et al. 2015), with uptake correlating with cognitive impairment (Chien et al. 2013). Moreover, a study showed tau signal increasing over 18 months in MCI and AD as symptoms progress (Mintun et al. 2015). In MCI and healthy subject’s association cortex tau binding was higher in amyloid-positive than amyloid-negative individuals (Pontecorvo et al. 2015). Regional tau correlated with mean cortical PIB binding in healthy controls, AD and MCI (Johnson et al. 2016). A [^11^C]PBB3 PET study also showed signal retention in the hippocampus in AD which spreads to association cortex as disease progresses (Maruyama et al. 2013). PET with [^18^F]AV1451 showed increased binding in MCI compared to controls in association cortical areas (Johnson et al. 2014). Therefore, tau PET imaging could provide a valid tool to monitor disease progression and cognitive decline in AD.

To understand the relationship between tau and amyloid-β pathology, tau deposition in healthy cognitively normal subjects has also been investigated with tau PET imaging. Cognitively normal subjects showed increasing accumulation of tau in basal ganglia, hippocampus, midbrain and fornix with ageing (Schultz 2015). Moreover, amyloid-β load and tau binding was observed in the inferior temporal lobe as well as an association between tau binding and rate of amyloid-β accumulation. These findings are consistent with histological findings that amyloid-β pathology can influences tau spreading throughout the cortex (Schultz 2015). It has been suggested that hippocampal tau is an age-related process, independent of AD but amplified by amyloid precursor protein dysfunction (Delacourte et al. 2002; Crary et al. 2014). Further studies combining tau and amyloid-β PET imaging could help to confirm whether the accumulation of amyloid-β triggers and accelerates the spread of tau deposition outside the mesial temporal cortex.

Preliminary results from [^18^F]PI-2620 demonstrate high signal in brain regions of known tau pathology in AD and distinct uptake patterns in PSP compared to AD (Barret et al. 2017). A pilot study with [^18^F]MK-6240 in four healthy controls and five AD patients, showed high uptake of [^18^F]MK-6240 in regions associated with NFT deposition in AD and negligible tracer binding in healthy individuals (Lohith et al. 2017).

### Tau imaging in parkinsonian dementia

Tau PET imaging in has the potential to differentiate DLB from PD and other parkinsonian tauopathies, due to patterns of tau disruption, and shed light on how tau aggregates contribute to cognitive decline in these disorders. To date, PET imaging has been employed to investigate tau deposition in DLB, PSP and CBS. Preliminary studies in PSP and CBS show [^11^C]PBB3 uptake in neocortical and subcortical structures in CBS patients (Shimada et al. 2015). This is suggestive that PBB3 might bind to non-AD tau conformations. However, a radiolabelled metabolite of PBB3 also crosses the blood–brain barrier making quantification less reliable. Therefore, the development of more selective derivatives is required.

While *in vitro* studies have shown [^18^F]AV1451 binds predominately to paired helical filament (PHF) of 3R-tau (Marquie et al. 2015) in AD brains, autoradiography studies in post-mortem CBS brains show low, but specific, [^18^F]AV1451 binding (Marquie et al. 2015; Sander et al. 2016; Lowe et al. 2016). Recently, [^18^F]AV1451 PET studies have shown tracer retention patterns in CBS patients matching the known distribution of tau pathology with high uptake in frontal and parietal cortical regions (Cho et al. 2017; Smith et al. 2017) (Fig. 1). Furthermore, increased uptake of [^18^F]AV1451, in the absence of amyloid, was shown to correlate with post-mortem levels of 4R-tau burden in two autopsy-confirmed CBS patient (Josephs et al. 2016; McMillan et al. 2016). In CBS patients [^18^F]AV1451 uptake was higher contralateral to the side with greater cortical dysfunction and parkinsonism (Cho et al. 2017; Smith et al. 2017). PET with [^18^F]THK5351 has also been studies to detect tau pathology in CBS. [^18^F]THK5351 uptake was higher in frontal, parietal and the globus pallidus in five CBS patients compared to healthy controls, with higher binding contralateral to the clinically most affected side (Kikuchi and Neurology 2016). Therefore, indicating the usefulness of [^18^F]AV1451 and [^18^F]THK5351 PET as markers to detect tau pathology in CBS.

Since [^18^F]AV1451 does not bind to Lewy bodies or TDP43 aggregates (Gomperts et al. 2015), it has been used to investigate tau levels in PSP and CBS. In PSP, increased [^18^F]AV1451 uptake has been reported in the striatum, pallidum, thalamus and frontal cortex (Whitwell et al. 2017; Passamonti et al. 2017). A study comparing uptake patterns of [^18^F]AV1451 in PSP and PD report distinct subcortical binding patterns, with greater subcortical binding in PSP and lower binding in the substantia nigra in PD compared to controls; however, there was no correlation between [^18^F]AV1451 binding and severity of motor dysfunction and no increased binding in cortical regions in PSP. Therefore, indicating [^18^F]AV1451 PET might not be optimal for assessing tau pathology in vivo in PSP. Preliminary results from the second generation tau tracer [^18^F]PI-2620 showed different and specific uptake patterns in AD and PSP, with no off-target binding, indicating its potential to aid differential diagnosis (Mueller et al. 2017; Barret et al. 2017).

Recently, two studies shave investigated patterns of tau distribution have been in patients with DLB (Gomperts 2016; Kantarci et al. 2017). Increased [^18^F]AV1451 uptake has been reported in the posterior temporoparietal, occipital cortex and precuneus in DLB compared to healthy controls (Gomperts 2016; Kantarci et al. 2017). Increased [^18^F]AV1451 uptake in the medial temporal cortex was able to distinguish AD dementia from probable DLB (Kantarci et al. 2017). Furthermore, a cross-sectional studies have compared [^18^F]AV1451 and [^11^C]PIB uptake in patients with DLB compared to PD with cognitive impairments (CI) and cognitively normal PD patients (Gomperts 2016), and patients with AD (Kantarci et al. 2017). In patients with DLB and PD with cognitive impairment, increased cortical [^18^F]AV1451 uptake, particularly in inferior temporal and precuneus regions, was observed compared to uptake in healthy controls (Gomperts 2016). Whereas PD patients without cognitive impairment did not show increased tau depositions compared to healthy controls. Increased tau deposition was observed in DLB patients with low cortical amyloid burden (Gomperts 2016). The presence of tau deposits with minimal amyloid load, indicated tau pathology is possible without amyloid in DLB. Furthermore, increased [^18^F]AV1451 uptake, but not [^11^C]PIB retention, in the inferior temporal gyrus and precuneus correlated with greater cognitive impairment in DLB and PD patients with cognitive impairment (Gomperts 2016).

### Tau imaging in frontotemporal lobar degenerative disorders

In FTD, PET with [^18^F]AV1451 has shown tracer binding in frontal, insular and anterior temporal cortex (Raboinovici et al. 2015). A single-case study of a patient with behavioural variant FTD showed increased [^18^F]AV1451 in the anterior temporal lobes and anterior cingulate cortex (Bevan Jones et al. 2016), regions known for tau accumulation in FTD (Kertesz et al. 2005). Furthermore, recently [^18^F]AV1451 PET has been shown to characterise tau pathology in a patient with FTD associated with the microtubule-associated protein tau (MAPT) mutation. [^18^F]AV1451 uptake was greatest in frontal, temporal and cingulate regions, and cortical binding correlated with cortical brain atrophy (Spina et al. 2017). Increased [^18^F]FDDNP uptake has also been reported in frontal and lateral temporal regions in FTLD compared to controls, similar to uptake observed in AD; however, in FTLD uptake in the parietal cortex is lower than in AD (Small et al. 2006). Therefore, [^18^F]FDDNP and [^18^F]AV1451 PET could provide a tool for evaluating the presence of tau deposits in FTD and to aid the differential diagnosis of FTLD from AD.

## Alpha-synuclein imaging

Alpha-synuclein is the defining component of Lewy bodies which are characteristic of PDD, DLB and FTD. Alpha-synuclein is an important target to understand the pathophysiology of neurodegenerative dementias and is a potential treatment target. The presence of α-synuclein has been implicated in mitochondrial and proteasomal dysfunction and vesicle trafficking within presynaptic dopaminergic neurons (Burre et al. 2010).

Alpha-synuclein is an amyloidogenic protein; therefore, PET tracers such as [^11^C]PIB and [^18^F]BF227 originally developed to target amyloid-β plaques have been investigated for their potential affinity to α-synuclein. There is some evidence of PIB binding to aggregated α-synuclein, its binding affinity was much higher for amyloid-β plaques. Moreover, preclinical PET data yielded inconsistent findings (Maetzler et al. 2008; Ye et al. 2008). Immunohistochemical studies demonstrated [^18^F]BF227 binding to Lewy bodies in PD brain sections (Fodero-Tavoletti et al. 2009). Recently, a novel α-synuclein tracer [^123^I]SIL23 (Bagchi et al. 2013) has been developed which binds to α-synuclein fibrils in post-mortem PD brain tissue. The distribution of SIL23 correlated with known levels of fibrillary α-synuclein in PD. Unfortunately, high non-specific binding in white matter limits the use of [^123^I]SIL23 in imaging studies.

To-date all tested compounds bind to other molecules, most commonly amyloid-β, and are, therefore, not suitable as selective tracers for the specific in vivo quantification of α-synuclein pathology. Work is on-going to generate a viable radiotracer for detecting α-synuclein *in vivo* which could serve to aid earlier and accurate diagnosis, and enable disease monitoring of future therapeutics.

## Cholinergic PET imaging

The cholinergic system is hypothesised to play a key role in cognitive processing and has a pivotal role in pathophysiology of dementias (Berger-Sweeney 2003). PET imaging has been employed to understand pre- and post-synaptic cholinergic dysfunction in dementia spectrum disorders, using markers for presynaptic choline acetyltransferase (ChAT) and acetylcholinesterase (AChE), and post-synaptic muscarinic acetylcholine receptors (mAChR) and nicotinic acetylcholine receptors (nAChRs) (Roy et al. 2016).

### Cholinergic imaging in Alzheimer’s disease

The cholinergic hypothesis states that degeneration of cholinergic neurons and subsequent decreased cholinergic neurotransmission leads to several cognitive and functional impairments in AD (Bartus et al. 1982). PET studies with [^11^C]PMP, a selective tracer for AChE (Irie et al. 1994), have shown reduced AChE activity (Kuhl et al. 1999) in the cortex, hippocampus and amygdala consistent with widespread ChAT and AChE loss observed in AD post-mortem studies (Perry et al. 1977). Decreased AChE was associated with cognitive impairments and in particular with functions of attention and working memory, instead of episodic memory, in AD (Bohnen et al. 2005). In APOE ε4 carriers, [^11^C]MP4A PET showed lower reductions in cortical AChE activity compared to non ε4 carriers (Eggers et al. 2006) suggesting APOE4 allele provides a protective role against widespread AChE activity loss. However, potential mechanisms underlying this are unclear. Recent development of the PET tracer [^18^F]FEOBV provides a promising tool to further assess the role of presynaptic vesicular acetylcholine transport (VAChT) dysregulation in AD (Petrou et al. 2014).

PET tracers [^18^F]2FA, targeting α_4_β_2_ nAChR and [^11^C]nicotine have been employed to investigate post-synaptic cholinergic dysfunction in AD (Sabri et al. 2008). PET with [^18^F]2FA showed significant reductions in nAChR availability in frontal, parietal and temporal cortices and the hippocampus in AD which correlated with cognitive decline (Sabri et al. 2008). Moreover, loss of nAChR was also observed in amnestic MCI in medial temporal cortex (Terriere et al. 2010), indicating PET with [^18^F]2FA may provide a tool for predicting conversion from MCI to AD. However, other studies have reported preservation of nAChRs in AD or MCI compared to healthy controls (Mitsis et al. 2009). Therefore, further studies are required to fully understand how early in the disease process cholinergic dysfunction occurs and the potential use for predicting conversion MCI to AD. Development of PET radioligands, such as [^18^F]AZAN, with faster regional kinetics than [^18^F]2FA, could aid further understanding of cholinergic dysfunction in AD (Horti et al. 2010). PET with [^11^C]nicotine has shown loss of nAChR binding in the frontal and temporal cortices and the hippocampus in moderate AD (Nordberg et al. 1995). As with presynaptic cholinergic markers, [^11^C]nicotine binding also correlated with attention deficit but not episodic memory or visuospatial impairment (Kadir et al. 2006). However, [^11^C]nicotine tracer has high levels of nonspecific binding and depends strongly on cerebral blood flow (Nyback et al. 1994).

Negative correlations have been reported between ChAT and nAChR availability and amyloid-β plaque load (Perry et al. 1977; Bierer et al. 1995; Okada et al. 2013); while reduction in AChE did not correlate with hypometabolism in posterior cingulate and parietal regions as measured with FGD-PET (Kuhl et al. 1999, 1996). Thus, suggesting increasing amyloid-β burden may induce degeneration of cholinergic neurons while different pathophysiological mechanisms could underlie cholinergic and metabolic decline in AD.

Acetylcholinesterase inhibitors are the main symptomatic treatment to improve cholinergic neurotransmission and cognitive function in AD (Declercq et al. 2016). PET studies before and after treatment with donepezil or rivastigmine, showed up to 40% frontal cortical inhibition of AChE activity. Clinical trials report modest improvement in behavioural and attentional symptoms in AD patients treated with AChE inhibitors (Kaasinen et al. 2002; Shinotoh et al. 2004). Following galantamine treatment reduced cortical [^11^C]PMP signal was observed indicating galantamine treatment increased ACh concentrations subsequently leading to increased cholinergic neurotransmission (Kadir et al. 2008). Clinical trials investigating the potential role of mAChR agonists/antagonists to ameliorate cognitive symptoms in AD are on-going (Declercq et al. 2016).

### Cholinergic imaging in parkinsonian dementias

In addition to loss of dopaminergic function other neurotransmitter systems, including cholinergic dysfunction, are involved in the pathophysiology of PD. Cholinergic PET imaging studies with [^11^C]MP4A or [^11^C]PMP uptake, has shown that loss of cortical AChE activity occurs early in PD and is more apparent in PDD than in non-demented PD (Bohnen et al. 2003; Hilker et al. 2005; Shimada et al. 2009). Lower cortical AChE activity was associated with reduced cognitive performance scores for attention, memory and executive functions (Bohnen et al. 2003) but not with severity of motor symptoms (Bohnen et al. 2006). This is consistent with post-mortem evidence suggesting loss of forebrain cholinergic function is associated with PDD (Hilker et al. 2005; Shimada et al. 2009; Bohnen et al. 2003). Loss AChE activity occurs early in PD becoming more widespread in PDD and DLB (Shimada et al. 2009). PDD patients exhibit higher cholinergic dysfunction than AD patients, matched according to degree of cognitive impairment, suggesting different mechanisms may underlie cognitive decline in PD and AD (Bohnen et al. 2003). Moreover, unlike in AD, thalamic AChE activity was decreased in PDD patients (Kotagal et al. 2012).

Differing cortical AChE activity in PSP and PD indicates potential use of cholinergic imaging markers in differential diagnosis (Gilman et al. 2010). Unlike in PD, PSP patients exhibit reduced thalamic [^11^C]MP4A uptake while striatal cholinergic neurons are unaffected (Mazere et al. 2012). CBS patients exhibit reduced AChE in paracentral, frontal, parietal and occipital cortices (Hirano et al. 2010). Combined, findings support the role of cholinergic dysfunction in PSP, CBS and PDD and potential therapeutic use of cholinesterase inhibitor (Matsunaga 2015).

### Cholinergic imaging in frontotemporal lobar degenerative disorders

PET studies showed no different in AChE activity in FTD patients compared to healthy controls (Hirano et al. 2010). This may explain why treatment with AChE inhibitors has proven ineffective (Mendez et al. 2007).

## Imaging neuroinflammation

Neuroinflammation and microglia activation plays a central role in the neurodegeneration process. Microglial activation is observed in numerous neurodegenerative dementias with differing protein pathologies, including amyloid, tau and alpha-synuclein, suggesting that microglia activation is not specifically related to one particular protein pathology, rather reflects a common neurodegenerative process. Understanding microglia activation in vivo will likely provide insights into pathophysiology of dementias, as well as a potential marker of disease progression.

Activated microglia cells overexpress mitochondrial translocator protein (TSPO) which can be targeted by PET radioligands (Leung 2004; Politis et al. 2012). The first generation TSPO tracer, [^11^C]PK11195 is most commonly used to image microglia activation and neuroinflammation in vivo. However, [^11^C]PK11195 has several limitations, such as high levels of non-specific binding, low brain penetration and poor signal-to-noise ratio (SNR) (Petit-Taboue et al. 1991; Boutin et al. 2007). Therefore, second generation TSPO radioligands were developed, including [^11^C]PBR28, [^11^C]DAA1106, [^11^C]DPA-713, [^18^F]DPA-714, [^18^F]FEDAA1106, [^18^F]FEPPA, [^18^F]PBR06 and [^18^F]PBR111, which have improve specificity, affinity and SNR (Chauveau et al. 2009). However, second generation radioligands have been found to be sensitive to a single nucleotide polymorphism in the TSPO gene (rs6971) which affects tracer binding affinity resulting in heterogeneity in quantitative PET data. Recently, novel third generation TSPO radioligands [^11^C]ER176 (Ikawa et al. 2017) and [^18^F]GE180 (Fan et al. 2016) have been developed as insensitive to rs6971 polymorphism. Preliminary results indicate low brain uptake of [^18^F]GE180 compared with [^11^C]ER176 (Fan et al. 2016). Furthermore, [^18^F]GE180 is yet to be investigated in low-affinity binders (LABs). In humans, [^11^C]ER176 has high sensitivity for all rs6971 genotypes [high- (HABs), mixed- (MABs) and LABs], therefore, allowing quantification of TSPO levels in LAB subjects (Ikawa et al. 2017). Therefore, [^11^C]ER176 could be a potential radioligand to quantify microglial levels in dementia disorders in vivo.

### Neuroinflammation in Alzheimer’s disease

Chronic neuroinflammation appears to play a central role in AD pathophysiology, and therefore, as a potential therapeutic target (Birch et al. 2014; Morales et al. 2014). PET studies with [^11^C]PK11195 show activated microglia in frontal, temporal, parietal, occipital and cingulate cortices in AD, with similar distribution pattern to that of amyloid- plaques (Edison et al. 2008). Moreover, increased cortical [^11^C]PK11195 binding was detected in about 60% of mild-to-moderate AD patients and 40% of MCI subjects (Cagnin et al. 2006). Detection of microglial activation in MCI suggests potential role for anti-inflammatory therapies in early disease stages (Okello et al. 2009). Association between the increased cortical [^11^C]PK11195 binding and MMSE suggests microglia activation is involved in neuronal dysfunction and cognitive impairment (Edison et al. 2008). However, not all studies detected increased [^11^C]PK11195 in MCI and mild-to-moderate AD but instead reported microglial activation limited to later stages of severe AD (Wiley et al. 2009). Discrepancies between results could be due to insensitivity of [^11^C]PK11195 to detect changes in microglia activation in mild-to-moderate AD, especially in studies with small sample sizes.

Second generation TSPO PET tracers, [^11^C]DAA1106 (Maeda et al. 2004), [^18^F]FEDAA1106 (Wang et al. 2012) have been used to assess microglia activation in AD (Yasuno et al. 2008). However, the affinity of these new tracers is dependent on which genetic polymorphism of TSPO is expressed (Kreisl et al. 2013). For example, binding to TSPO correlated with worse disease severity in AD (Kreisl et al. 2013). In AD patients without the rs6971 polymorphism, increased cortical [^11^C]PBR28 binding to TSPO has been reported but this cortical increase was not observed in MCI patients (Kreisl et al. 2013). Future studies with third generation PET tracers, such as [^11^C]ER176, which are insensitive to rs6971 polymorphism could provide further insights to the role of neuroinflammation in AD pathophysiology and monitor effective of potential disease-modifying therapies.

Neuroinflammation was also associated with increased accumulation of amyloid, measured with PET [^11^C]PIB, in AD and MCI (Kreisl et al. 2013). PET with [^11^C]PK11195 and [^18^F]FDG demonstrated significant inverse correlation between levels of microglial activation and cerebral glucose metabolic rate in the temporoparietal cortical regions (Fan et al. 2015). Thus, suggesting microglia activation has a detrimental effect on neuronal function. Microglia activation alongside amyloid deposition could cause neuronal damage and reduced glucose metabolism; while microglial activation and neuroinflammation could occur independently of amyloid deposition.

### Neuroinflammation in Parkinsonian dementias

[^11^C]PK11195 PET in PDD has shown increased levels of microglia activation in the association cortex compared to non-demented PD patients, consistent with pathological studies showing involvement of the association cortex in PD and DLB (McGeer et al. 1993; Imamura et al. 2003; Iannaccone et al. 2013). In PDD, increased microglia activation has been reported in the cingulate, frontal, temporal and occipital cortical regions, as well as the striatum compared to healthy controls (Edison et al. 2013). Microglia activation also correlated with cognitive impairment; thus, microglial activation could be a factor driving disease progression in PDD. Association between microglia activation and hypometabolism in the superior frontal, superior and medial temporal, parietal and occipital lobes has been reported in PDD suggesting microglial activation is co-localised with hypometabolism (Fan et al. 2015).

### Neuroinflammation in frontotemporal lobar degenerative disorders

Following recent discoveries of genes related to microglial activation, such as triggering receptor expressed on myeloid cells 2 (TREM2), in FTD (Rayaprolu et al. 2013) neuroinflammation is thought to play a role in FTD (Miller et al. 2013; Zhang 2015). Increased [^11^C]PK11195 binding in FTLD in the frontotemporal regions, suggests the presence of microglia reflecting progressive neuronal degeneration (Cagnin et al. 2004). A [^11^C]PK1195 PET study demonstrated microglial activation was higher in the temporal cortex in AD compared to FTLD, and greater in frontal subcortical white matter in FTLD compared to AD (Lant et al. 2014). This study showed higher temporal subcortical white matter in inherited FTLD with intronic mutations in MAPT compared to other genetic or non-genetic forms of FTLD (Lant et al. 2014). Thus, microglial involvement could serve as an in vivo marker of inherited FTLD–MAPT.

There are limited in vivo PET studies using second-generation TSPO PET tracers in FTD. A study using [^11^C]DAA1106 PET in pre-symptomatic MART gene carriers with parkinsonism linked to chromosome 17 (FTDP-17) showed increased microglial activation in frontal cortex compared to controls (Miyoshi et al. 2010). However, such increases were not overt throughout the brain in FTD. Further studies are required to track neuroinflammation in FTD and assessing the accuracy of TSPO imaging in detecting neuroinflammation in FTD. Early detection of neuroinflammation in FTD could identify novel treatment targets and tools for diagnosis and monitoring therapeutic efficacy.

## Future directions and conclusion

Synaptic dysfunction and loss of synaptic proteins is thought to play an importance role in AD where synaptic loss is correlated with severity of dementia (Davies et al. 1987; Terry et al. 1991). However, the role of synapse loss in other types of dementia is less well established (Clare et al. 2010). Synaptic decline could be a common feature in pathological cascade associated with dementia as well as other neurodegenerative disorders. Therefore, the development of new PET tracers targeting specific synaptic proteins, such as [^11^C]UCB-J tracer for synaptic vesicle glycoprotein 2A (SV2A) (Finnema et al. 2016), could help to understand how early synaptic dysfunction occurs and its role in symptomatic development in different dementia disorders.

Astrocytes are a key component of the neuroinflammatory response. Activated following stimulation with proinflammatory mediators, astrocytes increase in proliferation, cell hypertrophy, and astroglial markers. One of these markers is the glial fibrillary acidic protein (GFAP), which is regulated by Imidazoline 2 binding sites (I_2_BS) (Maragakis and Rothstein 2001; Volterra and Meldolesi 2005). Specifically, I_2_BS are expressed selectively on astrocyte mitochondrial membranes and upregulation may play a fundamental role in the biology of reactive gliosis (Tyacke 2012; Garcia-Sevilla et al. 1998). Early pre-clinical data that have suggested the link between I_2_BS and neuroinflammation (Head and Mayorov 2006). Glial cells also protect against excitotoxicity by clearing excess glutamate from the extracellular space (Maragakis and Rothstein 2001). This protective function may have relevant to the selective neurodegeneration in dementia spectrum disorders. The I_2_BS are mainly located on astrocytes in the cortex, hippocampus, basal ganglia and brainstem (De Vos et al. 1994). Recently, the PET radioligand [^11^C]BU9908 has been developed for in vivo quantification of I_2_BS showing high specificity and selectivity (Tyacke 2012; Kealey et al. 2013; Parker et al. 2014). PET studies evaluating [^11^C]BU99008 expression could highlight role of astroglial activation in disease progression and associations with symptomatology.

Over the last decade, PET imaging has provided key insights into neurodegenerative dementia disorders, helped to identify therapeutic targets and monitor the effects of treatments. PET imaging has identified molecular changes at early and preclinical disease stages so as to monitor disease progression and predict timings of symptomatic conversion. Moreover, our understanding of how patterns of cognitive dysfunction, overlapping and distinct changes in dementia disorders has increased over the last decade. Continued advances in PET imaging techniques and the development of new tracers are crucial to understand pathophysiological changes and provide markers to accurately differential between dementia types. MRI and PET are likely to be most powerful when used in combination both for research and translation into clinical practice. In the next decade, advanced MRI techniques and PET tracers for tau, α-synuclein and TDP-43 proteinopathies will likely play a central role in further understanding pathophysiology associated with dementia spectrum disorders. Moreover, the development of reliable in vivo imaging tools could facilitate the development of new disease-modifying therapies and provide clinical diagnostic biomarkers.
